# The interplay between the circadian clock and abiotic stress responses mediated by ABF3 and CCA1/LHY

**DOI:** 10.1073/pnas.2316825121

**Published:** 2024-02-06

**Authors:** Tong Liang, Shi Yu, Yuanzhong Pan, Jiarui Wang, Steve A. Kay

**Affiliations:** ^a^Department of Neurology, Keck School of Medicine, University of Southern California, Los Angeles, CA 90089

**Keywords:** circadian clock, abiotic stress, ABF, seed germination

## Abstract

Climate change is a global concern for all life on our planet, including humans and plants. Plants’ growth and development are significantly affected by abiotic stresses, including adverse temperature, inadequate or excess water availability, nutrient deficiency, and salinity. The circadian clock is a master regulator of numerous developmental and metabolic processes in plants. In an effort to identify new clock-related genes and outputs through bioinformatic analysis, we have revealed that CIRCADIAN CLOCK ASSOCIATED 1 (CCA1) and LATE ELONGATED HYPOCOTYL (LHY) play a crucial role in regulating a wide range of abiotic stress responses and target *ABSCISIC ACID RESPONSIVE ELEMENTS-BINDING FACTOR3* (*ABF3*), a key transcription factor in the plant hormone Abscisic acid (ABA)-signaling pathway. Specifically, we found that CCA1 and LHY regulate the expression of *ABF3* under diel conditions, as well as seed germination under salinity. Conversely, ABF3 controls the expression of core clock genes and orchestrates the circadian period in a stress-responsive manner. ABF3 delivers the stress signal to the central oscillator by binding to the promoter of *CCA1* and *LHY*. Overall, our study uncovers the reciprocal regulation between ABF3 and CCA1/LHY and molecular mechanisms underlying the interaction between the circadian clock and abiotic stress. This finding may aid in developing molecular and genetic solutions for plants to survive and thrive in the face of climate change.

## Significance

Climate change poses a global threat to plants and humans. Understanding how plants respond to abiotic stresses is crucial for addressing this challenge. Here, we reveal the important role of the circadian clock in regulating abiotic stress responses through the reciprocal regulation between CIRCADIAN CLOCK ASSOCIATED 1 (CCA1) and LATE ELONGATED HYPOCOTYL (LHY) and ABSCISIC ACID RESPONSIVE ELEMENTS-BINDING FACTOR3 (ABF3). We demonstrate how the circadian clock influences *ABF3* expression, which in turn delivers stress signals to core clock genes and adjusts the circadian period in response to stress. These findings offer valuable insights for developing genetic and molecular approaches to enhance plant resilience in the face of climate change.

As sessile organisms, plants must constantly adapt to the environment and cope with various stresses, such as drought, salinity, and extreme temperatures. Plants have evolved various mechanisms to adapt to abiotic stress, and one of the most crucial mechanisms is the ABA signaling pathway. ABA is a plant hormone that plays a key role in regulating responses to abiotic stress such as drought, high salinity, and high temperatures (1, 2). When plants experience stress, ABA levels increase, and this activates the ABA signaling pathway, which comprises ABA receptor Pyrabactin Resistance (PYR) and PYR-Like (PYL), co-receptor PP2Cs (type 2C Ser/Thr protein phosphatases), SnRK2 kinases (SNF1-related protein kinases), ABI5 (ABA-Insensitive5), and ABA-responsive element binding factors (ABFs) (3–8). In response to increased ABA, PYR/PYL receptors interact with and inactivate PP2Cs, releasing the inhibition of SnRK2s kinases that promote the activation of downstream transcription factors ABI5 and ABFs. The ABA signaling pathway triggers a series of responses within the layers of cells, tissues, and whole plants to confer tolerance to stress (9). The ABA-responsive element (ABRE) is the major cis-element for ABA-responsive gene expression, and ABFs regulate ABRE-dependent gene expression (10, 11).

As the internal timekeeping machinery, the circadian clock enables plants to synchronize with daily and seasonal environmental changes and directly controls many developmental processes throughout the life cycle (12, 13). The circadian clock in Arabidopsis includes a plethora of oscillating proteins that are expressed and function in an orchestrated manner (14). The morning-expressed transcription factors CIRCADIANCLOCK ASSOCIATED 1 (CCA1) (15) and LATEELONGATED HYPOCOTYL (LHY) (16) repress the expression of the evening-expressed TIMING OF CAB EXPRESSION 1 (TOC1) (17), which establishes the core negative feedback loop of the circadian oscillator (18, 19). In addition to TOC1, other members of the pseudo-response regulator (PRR) protein family PRR9/PRR7/PRR5, expressed sequentially throughout the day, form an additional loop (20–22). The Evening complex (EC) composed of EARLY FLOWERING 3 (ELF3), ELF4, and LUX ARRHYTHMO/PHYTOCLOCK1 (LUX) (23) integrates ambient temperature signals and inhibits the expression of morning genes PRRs (24), and the expression of EC components are repressed by CCA1 and LHY (25, 26).

The circadian clock is involved in various abiotic stress responses. CCA1, LHY, and PRR play a role in cold responses by regulating the expression of cold-responsive genes C-repeat/DRE binding factor 1 (*CBF1*), *CBF2*, and *CBF3* (27, 28). Conversely, cold temperature regulates the alternative splicing of *CCA1* (28, 29) and CBF1 mediates cold input to the circadian clock by directly regulating the expression of *LUX* (30). Heat Shock Factor B2b (HsfB2b) and FLOWERING BASIC HELIX-LOOP-HELIX 1 (FBH1) can mediate the high-temperature signal to the circadian clock by directly targeting *PRR7* (31) and *CCA1* (32), respectively. Moreover, TOC1 binds the promoter of the *ABA-related gene* (*ABAR/CHLH/GUN5*) and controls the responses to drought (33). PRR9/PRR7/PRR5 gene mutations confer more tolerance to drought, high salinity, and cold (34), and PRR7 can directly regulate oxidative stress and stomata conductance (35). GIGANTEA (GI) can regulate drought tolerance by modulating ABA biosynthesis and stomatal closure (36). GI can also regulate salt tolerance by sequestering SALT OVERLY SENSITIVE 2 kinase (37), while ELF3 can enhance plants’ resilience to salt stress by suppressing GI (38). In addition to regulating stress signaling components, the circadian clock can regulate plant hormone biosynthesis, including ABA (39). ABA level accumulates rhythmically in Arabidopsis and LHY can directly control the genes responsible for ABA biosynthesis (40). Studies in crops also revealed the important role of the circadian clock in regulating stress responses. The circadian clock can gate genome-wide cold responses in bread wheat (41). PRR homologous gene Ppd-H1 in barley integrates drought stress signals to modulate spike development and flowering time (42). CCA1 and LHY orthologues in soybean can negatively regulate drought tolerance and repress ABA responses (43, 44).

CCA1 and LHY are two core circadian proteins and much transcriptional legacy data are available for their target gene regulations. In this study, we re-evaluated and mined the RNA-seq and ChIP-seq data of CCA1 and LHY with a modified bioinformatics pipeline to identify the direct targets of either CCA1 or LHY. This analysis revealed that CCA1 and LHY are involved in multiple types of abiotic stress responses. Specifically, CCA1 and LHY regulate the expression of *ABF3* (ABSCISIC ACID RESPONSIVE ELEMENTS-BINDING FACTOR3) and seed germination under salinity. Conversely, ABF3 regulates the expression of core clock genes and shortens the circadian period in a stress-responsive manner.

## Results

**Fig. 1.**
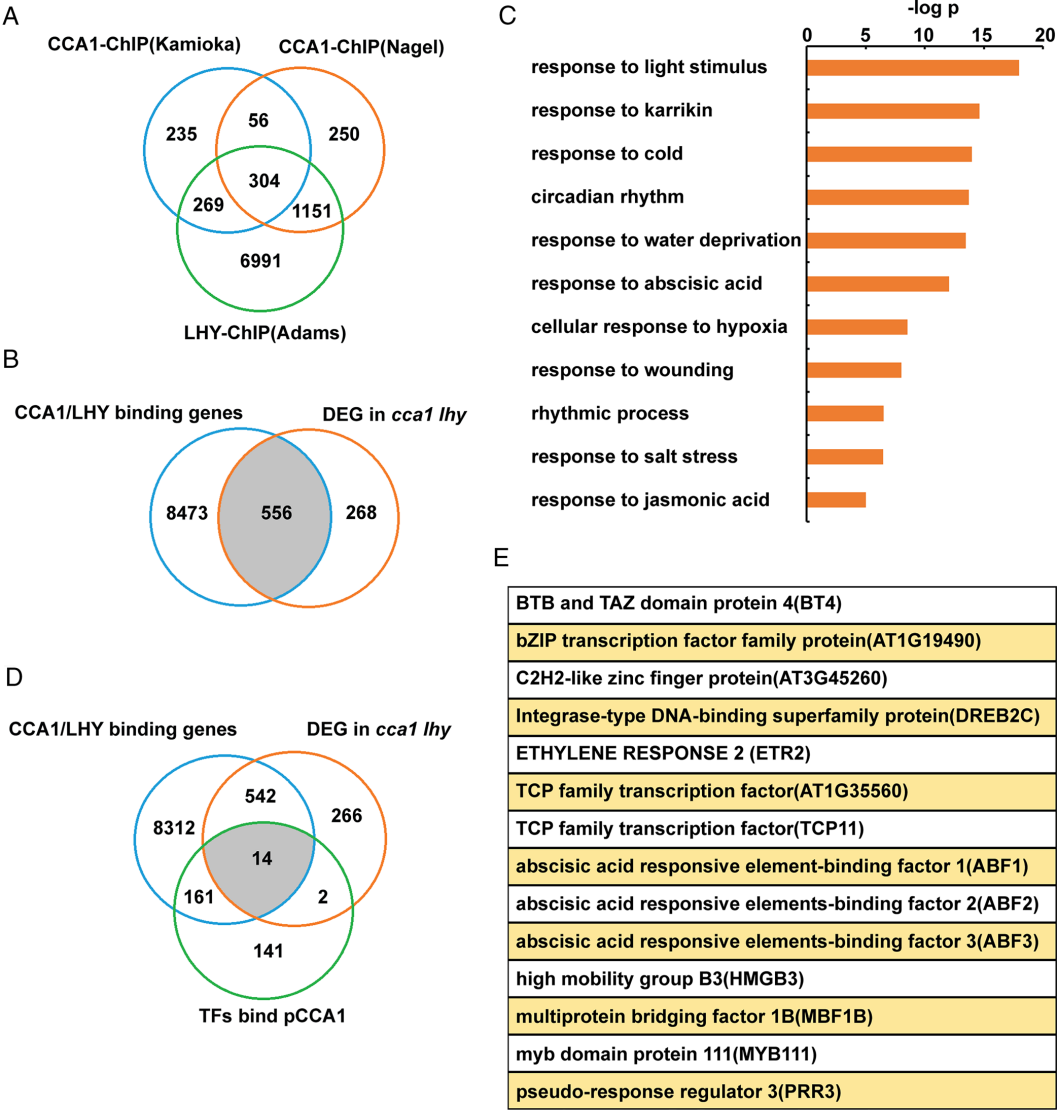
Transcriptomic analysis identified the reciprocal regulatory genes of CCA1 and LHY. (*A*) The ChIP-seq datasets from three research groups CCA1-ChIP(Nagel) and CCA1-ChIP(Kamioka) (45, 46) and LHY-ChIP(Adams) (40) were re-evaluated to characterize the genes bound by CCA1 or LHY. The Venn diagram shows the overlap of the three datasets. (*B*) The DEGs in *cca1 lhy* mutant (46) were overlapped with the combined dataset in *A*. (*C*) GO annotations of the 556 overlapped genes in *B*. Fold change log values are shown. (*D*) The Venn diagram shows the overlap of the ChIP-seq and RNA-seq in *B* with TFs binding *CCA1* promoter. (*E*) Gene names of the 14 overlapped genes shown in *D*.

The core clock genes CCA1 and LHY have been extensively studied, with a wealth of legacy data available. To further explore the roles of CCA1 and LHY and identify the output genes, we collected the ChIP-seq data of CCA1 and LHY from multiple studies (40, 45, 46) and conducted a re-evaluation of the data (Fig. 1*A*). The previous studies usually focused on the overlapping genes of multiple datasets, resulting in the identification of common targets of CCA1 and LHY or stringent target genes from multiple tests, such as the 304 genes in our case (Fig. 1*A*). However, this approach missed many genes that are specific targets of either CCA1 or LHY, as well as those that are conditionally regulated by CCA1 or LHY. So, we developed a unique procedure by combining the three ChIP-seq datasets and identified a total of 8,473 target genes of CCA1 or LHY. We then overlapped these target genes with the differentially expressed genes (DEGs) in the *cca1 lhy* mutant (46) to obtain 556 genes, which are likely to be regulated and targeted by either CCA1 or LHY (Fig. 1*B*). The GO annotations revealed some canonical regulations of CCA1 and LHY, such as response to light and circadian rhythm processes (Fig. 1*C*). Interestingly, we also observed that a variety of abiotic stress processes, including response to cold, water deprivation, wounding, and salt, were enriched in the gene annotations (Fig. 1*C*). So, our analysis pipeline effectively validated the known roles of CCA1 and LHY and revealed their putative involvement in multiple stress-related processes, with abiotic stress genes constituting significant outputs. To further explore the transcriptomic datasets, we utilized the yeast one hybrid (Y1H) screening data against the *CCA1* promoter (47), which was screened against our lab’s comprehensive transcription factor library. This led to the identification of 14 genes (Fig. 1 *D* and *E*), that are supposed to be output genes of CCA1 or LHY and can bind the promoter of *CCA1*. Notably, three ABFs, key transcription factors in the ABA signaling pathway, were among the 14 genes, consistent with our findings that CCA1 and LHY may be involved in multiple stress processes and suggest the potential role of ABFs in regulating the circadian clock.

**Fig. 2.**
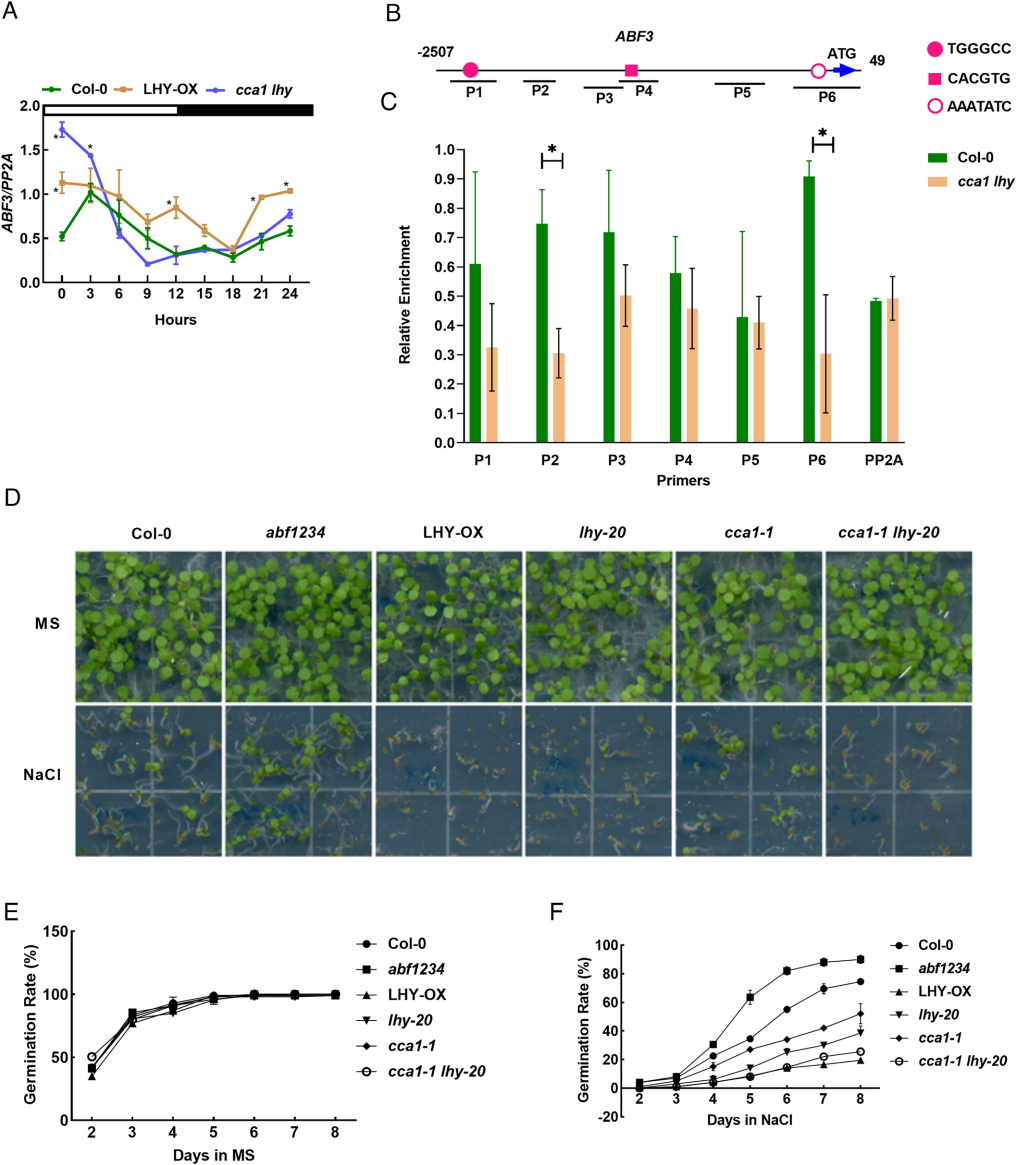
CCA1 and LHY regulate the expression of *ABF3* and seed germination. (*A*) RT-qPCR analysis of *ABF3* in Col-0, LHY-OX, and *cca1 lhy* in the diel condition. The seeds were grown in ½ MS under 12-h:12-h long-day cycles for 10 d and collected samples every 3 h as indicated in the diagram. The *PP2A* gene was analyzed as an internal control. Error bars, SDs of three biological replicates. *P < 0.05, compared to corresponding Col-0 of same time point, by *t* test using Excel. (*B*) Motif analysis of *ABF3* promoter. The upstream 2507bp of *ABF3* was under analysis. The red ball indicates TGGGCC, a TCP binding site. The red square indicates CACGTG, a G-box, and also a segment of ABRE. Red circles indicate AAATATC, a segment of EE. P1 to P6 indicate primer pairs used for ChIP-qPCR in *C*. (*C*) LHY binds to the promoter of *ABF3* in vivo. 12-d-old Col-0 and *cca1-1 lhy-20* (negative control) plants were harvested for ChIP assays. Chromatin fragments were immunoprecipitated by anti-LHY beads (IP) or without immunoprecipitation (input). The precipitated DNA was analyzed by RT-qPCR using primer pairs indicated in *B*. The level of binding was calculated as the ratio between IP and input, normalized to that of *ACT7* as an internal control. Error bars, SDs of three biological replicates. *P < 0.05, by *t* test using Excel. (*D*–*F*) Seed germination analysis of Col-0, *abf1234*, LHY-OX, *lhy-20*, *cca1-1,* and *cca1-1 lhy-20* under normal conditions (MS medium) or under salinity (MS medium containing 120 mM NaCl). Seed germination rates were quantified from the second day to the 8th day after sowing in the 16-h:8-h long day cycles (*E* and *F*). Error bars, SDs of three biological replicates, with at least 40 seeds per genotype in each replicate. A crop of the representative figures on the 6th day is shown in *D*.

Out of the four *ABFs* in Arabidopsis, the transcription of *ABF1* and *ABF3* exhibit significant oscillation in the diel conditions, with *ABF3* peaking in the morning and *ABF1* peaking near dusk (*SI Appendix*, Fig. S1*A*). The analysis of transcriptomic data has suggested CCA1 or LHY may regulate the expression of *ABF1* and *ABF3* (Fig. 1 *D* and *E*). RT-qPCR was performed to verify this regulation, and we found the expression of *ABF1* and *ABF3* are indeed altered in LHY-OX and *cca1-1 lhy-20* in the diel conditions (Fig. 2*A* and *SI Appendix*, Fig. S1*B*). We analyzed the promoter of *ABF3* and identified several binding sites of CCA1 and LHY, including G-box (CACGTG), Evening Element (EE) segment (AAATATC), and TCP-binding site (TGGGCC) (40) (Fig. 2*B*). ChIP (Chromatin immunoprecipitation) assays confirmed that LHY can bind the promoter of *ABF3* in vivo (Fig. 2*C*). It is well established that ABFs play a crucial role in mediating plant stress responses and are induced by abiotic stress (10). In order to investigate whether the stress responsiveness of *ABF3* is regulated by the circadian clock, Arabidopsis seedlings Col-0 (Columbia-0) were entrained in 12/12 long-day conditions for 8 d and then subjected to NaCl or ABA treatment at either ZT1 or ZT10 for a 2-h time course (*SI Appendix*, Fig. S1*C*). To ensure that all plants are under light conditions throughout the operation, the plant chambers were adjusted to constant light conditions since the day treatments were applied (*SI Appendix*, Fig. S1*C*). The expression of *ABF3* was induced by ABA both in the morning (ZT1) and afternoon (ZT10) (*SI Appendix*, Fig. S1*D*), and the induction rate was found to be higher at ZT10 than at ZT1 (*SI Appendix*, Fig. S1*E*). Similar to ABA, the induction rate of *ABF3* expression in response to NaCl treatment was slightly higher at ZT10 than at ZT1 (*SI Appendix*, Fig. S1 *F* and *G*). In summary, the induction rate of *ABF3* differs at ZT1 and ZT10, suggesting that the circadian clock may gate the stress responsiveness of *ABF3*.

The transcriptomic data have indicated a potential role for CCA1 and LHY in regulating various stress processes (Fig. 1 *A*–*C*). To confirm this hypothesis, we examined seed germination in response to salinity as the readout of stress responses. We tested various mutant lines in the Col-0 background, including ABFs knockout mutant *abf1234* (48), CCA1 knockout null mutant *cca1-1* (49), LHY knockdown mutant *lhy-20* (50), double mutant *cca1-1 lhy-20*, and LHY overexpression line LHY-OX, along with the wild type Col-0. Under normal conditions, all the tested lines germinated normally (Fig. 2 *D*–*F*). However, upon the treatment of NaCl, the germination rate of *abf1234* was higher than that of Col-0, whereas *cca1-1* and *lhy-20* exhibited compromised germination rates, and *cca1-1 lhy-20* double mutant had an additively lower germination rate (Fig. 2 *D*–*F*). Interestingly, LHY-OX also showed a similarly low level of germination rate as *cca1-1 lhy-20* double mutant.

**Fig. 3.**
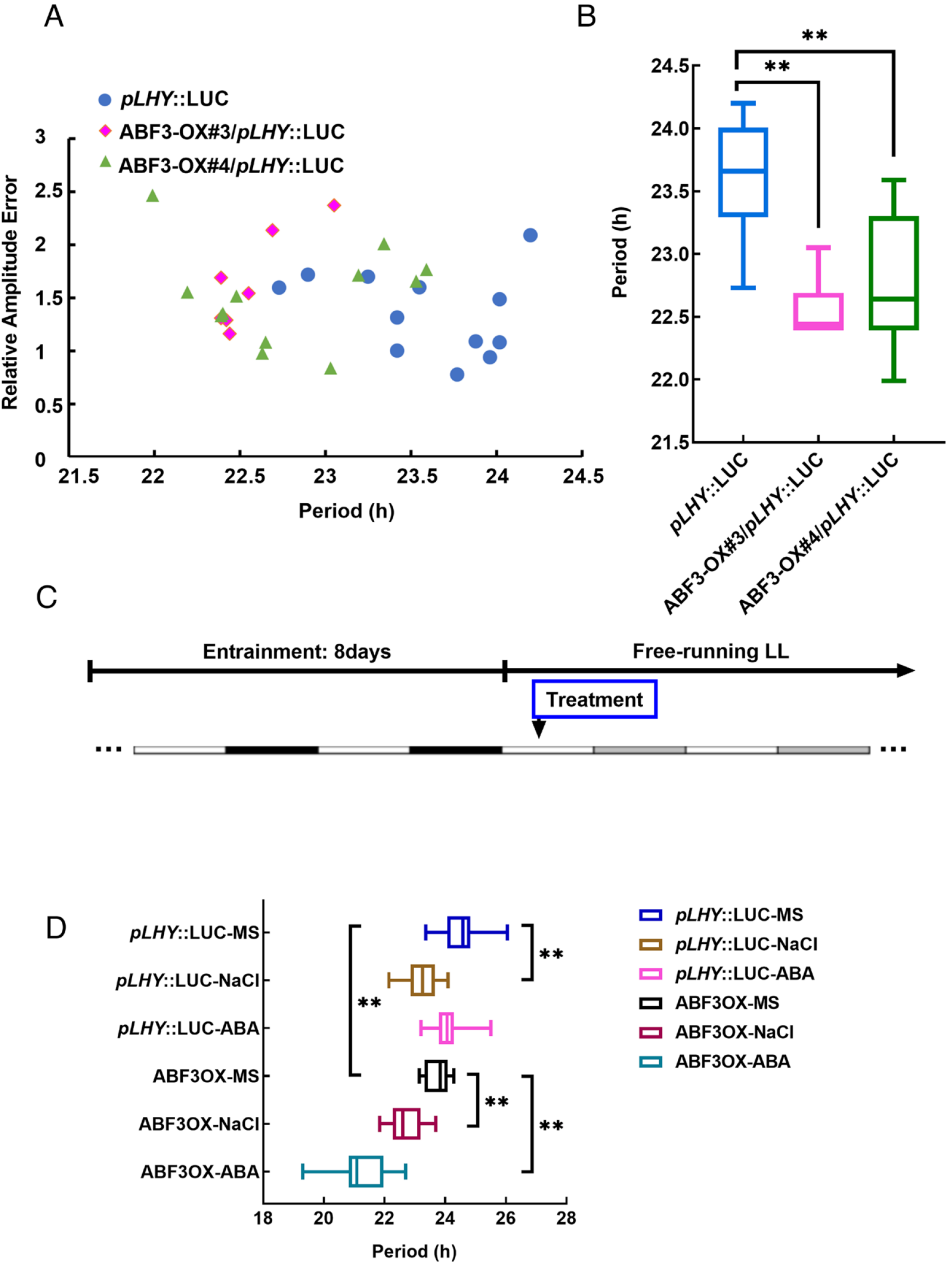
ABF3 regulates the circadian period in a stress-responsive manner. (*A* and *B*) Bioluminescence analysis of *pLHY*::LUC expression and ABF3-OX/*pLHY*::LUC. The indicated genotypes were grown in ½ MS under 12-h:12-h long-day cycles for 8 d, then released to free-running LL conditions and detected by a bioluminescence reader. Period and amplitude values were generated by FFT-NLLS. The quantified circadian period was shown (*B*). **P < 0.01, by *t* test using Excel. (*C*) The experiment schema for phenotyping the circadian period with stress treatment. Arabidopsis seedlings were entrained in ½ MS under 12-h:12-h long-day cycles for 8 d, then transferred to ½ MS medium containing 50 µM ABA or 120 mM NaCl before releasing them to LL condition. Then, 1 mM luciferin was added to the plants, and the luciferase signal was detected by a bioluminescence reader. (*D*) The circadian period of *pLHY*::LUC and ABF3-OX/*pLHY*::LUC, treated with MS medium control, or 50 µM ABA or 120 mM NaCl, as indicated in (*C*). Values are shown as means ± SEM; n = 12. **P < 0.01, by *t* test using Excel.

ABFs were identified as hits in the *CCA1* promoter Y1H screening (Fig. 1 *D* and *E*), so ABFs are likely to regulate the circadian clock. Due to the redundancy of ABFs, we overexpressed *ABF3* in a circadian clock reporter line *pLHY*::LUC, which works well to evaluate effects on the circadian clock. The overexpression of *ABF3* caused a significantly short period of *pLHY*::LUC (Fig. 3 *A* and *B* and *SI Appendix*, Fig. S2 *A* and *B*), indicating that ABF3 can regulate the circadian clock pattern in Arabidopsis. ABF3 is involved in various abiotic stress responses, such as drought and high salinity, and is a major transcription factor in the ABA signaling pathway (10, 48), so we explored whether abiotic stress is an upstream signal of ABF3 that promotes regulation of the circadian clock. We entrained Arabidopsis seedlings in 1/2 MS (Murashigeand Skoog) medium under 12/12 long-day conditions for 8 d and then transferred the plants to the medium containing ABA or NaCl or KCl in the free-running conditions to examine the circadian activity (Fig. 3*C*). For the *pLHY*::LUC reporter line, ABA treatment exhibited a minor impact on the circadian period, while NaCl or KCl treatment notably shortened the period compared to the control on MS medium (Fig. 3*D* and *SI Appendix*, Fig. S2*C*). When ABF3 was overexpressed in the *pLHY*::LUC line, exposure to NaCl treatment resulted in a further reduction in the circadian period compared to the MS medium control (Fig. 3*D*). Furthermore, ABA treatment in the ABF3-OX/*pLHY*::LUC line led to a significantly shorter period than in the same line without treatment, highlighting an increased sensitivity to ABA due to ABF3 overexpression.

**Fig. 4.**
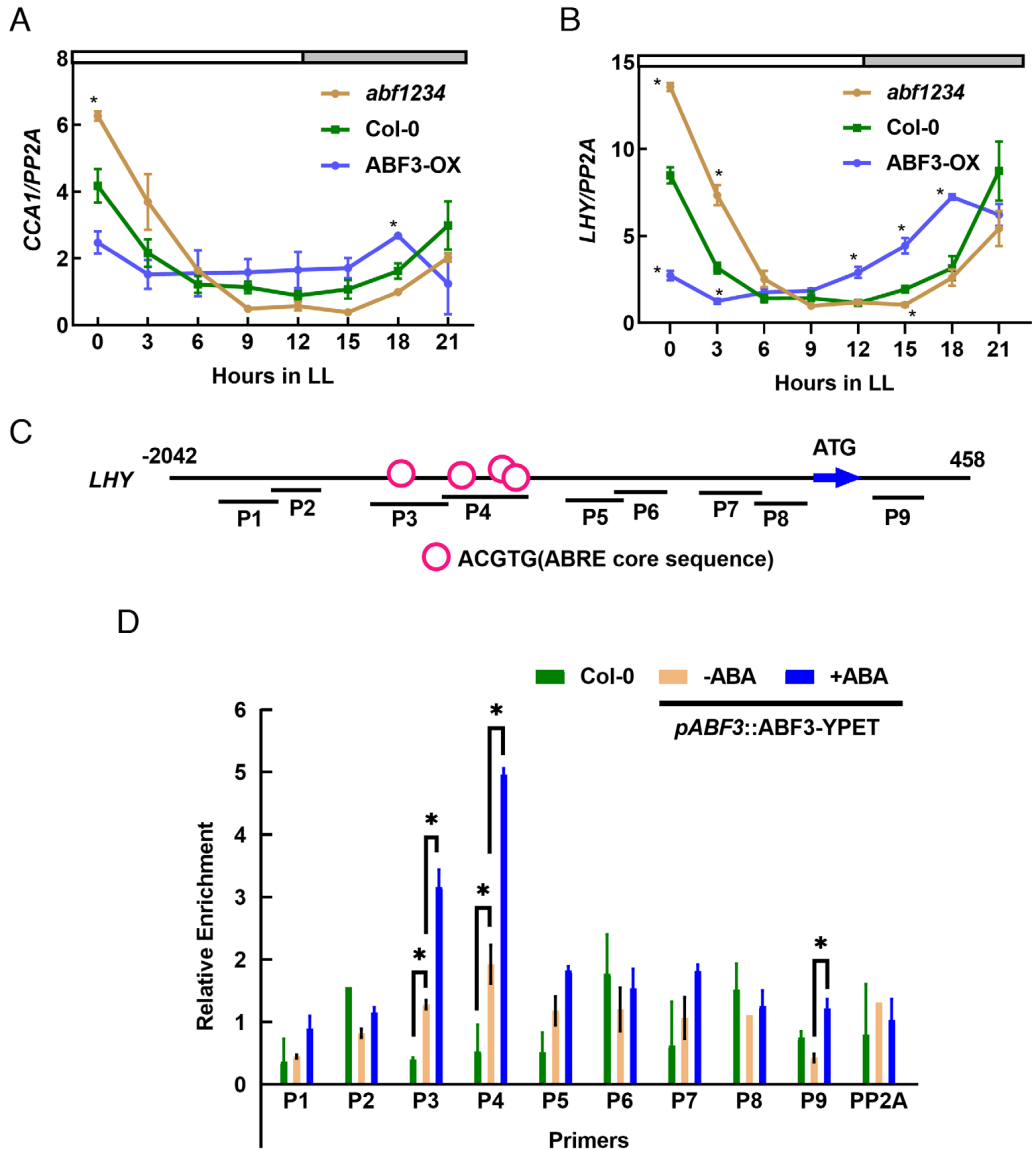
ABF3 regulates the expression of core clock genes. (*A* and *B*) RT-qPCR analysis of gene expression of *CCA1* (*A*), *LHY* (*B*) in Col-0, ABF3-OX and *abf1234*. The indicated genotypes were grown in ½ MS under 12-h:12-h long-day cycles for 10 d, then released to free-running LL conditions. The seedlings were harvested every 3 h, as indicated in the diagram. The *PP2A* gene was analyzed as an internal control. Error bars, SDs of three biological replicates. **P < 0.05, compared to corresponding Col-0 of same time point, by *t* test using Excel. (*C*) Promoter analysis of *LHY* promoter. Red circles indicate ACGTG (ABRE core sequence). P1 to P9 indicate primer pairs used for ChIP-qPCR. (*D*) ChIP results show that ABF3 binds to the promoters of *LHY* in vivo. 12-d-old Col-0 and *pABF3*::ABF3-YPET transgenic plants were treated with or without 1 μM ABA for 2 h before harvesting samples. Chromatin fragments were immunoprecipitated by anti-GFP beads (IP) or without immunoprecipitation (input). The precipitated DNA was analyzed by RT-qPCR using primer pairs indicated in *C*. The level of binding was calculated as the ratio between IP and input, normalized to that of *ACT7* as an internal control. Error bars, SDs of three biological replicates. *P < 0.05, by *t* test using Excel.

In light of ABF3 binding the *CCA1* promoter in the Y1H screening (Fig. 1 *D* and *E*), we conducted RT-qPCR with ABF3-OX and *abf1234* mutant to analyze gene expression of core clock genes. The Arabidopsis seedlings were grown in 12/12 long-day conditions for 10 d, and samples were harvested in free-running conditions for transcripts analysis. The expression patterns of *CCA1*, *LHY*, *TOC1*, and *PRR9* were altered in the ABF3-OX and *abf1234* mutant (Fig. 4 *A* and *B* and *SI Appendix*, Fig. S3 *A* and *B*). Specifically, the peaks of *CCA1* and *LHY* expression were down-regulated in ABF3-OX while up-regulated in *abf1234* mutant (Fig. 4 *A* and *B*). This is consistent with our findings and previous findings that ABF3-OX, *cca1,* and *lhy* mutant all have short circadian periods (49, 51). We also analyzed the promoters of *CCA1* and *LHY* and identified several predicted ABREs (Fig. 4*C* and *SI Appendix*, Fig. S3*C*). ABRE motifs are known to be bound by ABFs and critical for the expression of the ABA-responsive genes (10, 11). ABRE was also reported to be a clock-controlled promoter element (39). To determine whether ABF3 directly targets *CCA1* or *LHY*, we performed ChIP assays using *pABF3*::ABF3-YPET (52). The ChIP results showed that ABF3 could bind the promoters of both *CCA1* and *LHY* (*SI Appendix*, Fig. S3*D*). Moreover, we investigated whether the DNA binding activity of ABF3 is modulated by abiotic stress. We treated *pABF3*::ABF3-YPET transgenic lines with or without ABA before collecting samples for ChIP and analyzed them with a set of primers covering the promoter of *LHY* (Fig. 4 *C* and *D*). Compared to Col-0, ABF3 showed a high binding affinity to the promoter region containing ABRE segment, indicating ABF3 binds to the LHY promoter through ABRE motifs. Furthermore, the ABA treatment enhanced the DNA binding activity of ABF3, particularly in the regions containing the ABRE segment, indicating that the ABA signal can strengthen the binding activity of ABF3 toward the *LHY* promoter.

## Discussion

Studies have revealed the role of the circadian clock in regulating ABA responses and biosynthesis at the genome-wide level (28, 39, 53). Notably, TOC1 binds the promoter of *ABAR* and controls the responses to drought (33). CCA1 and LHY can directly target *CBF1* and regulate freezing tolerance (27). The direct targets of PRR7 include many genes involved in abiotic stress responses (35). Our study reveals a unique mechanism of the interplay between the circadian clock and abiotic stress responses mediated by ABF3 and CCA1/LHY (*SI Appendix*, Fig. S3*E*). We investigated the seed germination phenotype as the readout of stress responses. We demonstrate that core clock proteins CCA1 and LHY are involved in the seed germination rate under salinity and exert regulatory control over the expression of *ABF3* under diel conditions. Among CCA1 and LHY targets, evening-expressed genes are highly enriched, and many of them contain EE motifs; however, CCA1 and LHY can also target morning-expressed genes or arrhythmic genes (45) *ABF3*, featuring an oscillating pattern with a peak in the morning, falls in the category of CCA1/LHY targeted morning-expressed genes. Circadian clock can gate cold responses in Arabidopsis and bread wheat (41, 54), specifically low-temperature induction of *CBF1, 2,* and *3* is gated by the circadian clock in Arabidopsis (54). Here, we demonstrate that the stress-induction of *ABF3* differs at ZT1 and ZT10, suggesting the circadian clock may gate the stress responsiveness of *ABF3*. However, more time points throughout the circadian cycle are needed to fully understand the circadian gating mechanism. Moreover, we observed that LHY directly binds to the promoter region of *ABF3* (Fig. 2*C*), further supporting its role in the modulation of *ABF3* expression. The regulation of *ABF3* by CCA1 and LHY provides a mechanistic explanation for how the circadian clock components modulate abiotic stress responses (*SI Appendix*, Fig. S3*E*).

The process of seed germination is controlled by plant hormones and external environmental signals, such as water, cold temperatures, and light (55–58). It is worth noting that the circadian clock exists in the very early development, even days after seed imbibition without entraining light or temperature (59). The clock machinery plays a significant role in regulating seed dormancy and germination, particularly in response to low temperatures and dry after-ripening (60). In our study, we utilized seeds from the Col-0 background that had undergone a dry after-ripening period of 3 wk. We observed that *cca1* and *lhy* mutants, as well as LHY-OX plants, exhibited similar germination rates to Col-0 under 22 °C conditions. However, these seeds in Ws background, freshly harvested or after-ripened for 3 mo, showed germination variations under different temperature conditions (60), suggesting that the context of different genetic background and seed age may influence the circadian clock’s role in regulating seed germination in response to temperature cues. Interestingly, both *cca1 lhy* and LHY-OX showed lower germination rates than Col-0 under salinity (Fig. 2 *D*–*F*). Although there were altered expressions of *ABF1* and *ABF3* in both *cca1 lhy* and LHY-OX (Fig. 2*A* and *SI Appendix*, Fig. S1*B*), it’s unclear whether these changes solely contribute to the observed seed germination phenotype. As Fig. 1 indicates, CCA1 and LHY regulate numerous stress-related genes, many of which influence seed germination. Hence, the germination phenotype in *cca1 lhy* and LHY-OX might be attributed to multiple factors. Other circadian clock components like GI, PRR7, TOC1, and ZTL also play roles in seed germination (60–62). Mutations or overexpression of CCA1/LHY could disrupt the circadian feedback loop, leading to unexpected phenotypes. To unravel this complexity, we need to further analyze the system-wide expression of a broader range of stress-related and seed germination-associated genes in *cca1 lhy* and LHY-OX.

Our research also uncovers a unique function of ABF3 in modulating the circadian clock. ABF3 controls the expression of core clock genes and modulates the circadian period in a stress-responsive manner (Figs. 4 *A* and *B* and 3*D* and *SI Appendix*, Fig. S3 *A* and *B*). Specifically, ABA treatment enhanced ABF3 binding to the promoter of *LHY* (Fig. 4*D*), possibly by inducing the phosphorylation of ABF3 and activating its DNA binding activity. ABF3 can bind to the promoter of *LHY* in a stress-enhanced manner, delivering the stress signal to the central oscillator and modulating the expression of core clock genes (*SI Appendix*, Fig. S3*E*). This reciprocal regulation between ABF3 and LHY establishes a dynamic feedback loop that enables plants to adapt and fine-tune responses to abiotic stress and promote resilience in changing environmental conditions.

Besides regulating the transcripts of ABA signaling factors, LHY can also modulate the internal ABA level by directly regulating the expression of the ABA-biosynthesis genes (40), indicating that the interplay between the circadian clock and abiotic stress occurs at multiple levels and through various mechanisms. Consistent with previous studies that exogenous ABA treatment had a minor effect on the circadian clock period (63), we observed a barely changed circadian period of *pLHY*::LUC with ABA treatment alone (Fig. 3*D*). We speculate that the LHY regulation of internal ABA biosynthesis buffers the effect of the exogenous ABA treatment. However, ABF3 overexpression caused a short period of *pLHY*::LUC, indicating that the key factor of the ABA signaling pathway is still involved in circadian clock regulation. Different from ABA, the treatment of KCl or NaCl caused a short period of *pLHY*::LUC (*SI Appendix*, Fig. S2*C*). High salt concentration imposes both hyperosmotic and hyperionic stress on plants. More effort is needed to dissect which factor is responsible for the salinity-specific phenotype and explain the discrepancy between ABA and salinity. Interestingly, ABF3-OX/*pLHY*::LUC under stress treatment, particularly under ABA treatment, exhibited a very short circadian period, indicating that ABF3-OX lines are hypersensitive to stress in regulating the circadian period.

In summary, by elucidating the regulatory pathways and feedback loops involving CCA1, LHY, and ABF3, we gain a deeper understanding of how plants coordinate their physiological and developmental responses to environmental challenges. Understanding the intricate relationship between the circadian clock and abiotic stress response at the molecular level may aid in the development of targeted strategies to enhance plant resilience in the face of climate change.

## Materials and Methods

### Plant Materials and Growth Conditions.

*Arabidopsis thaliana* Col-0 was used for most experiments unless otherwise indicated. The *cca1-1* in Col-0 (49), *lhy-20* in Col-0 (50), *abf1234* (48), *pLHY*::LUC (64), and *pABF3*::ABF3-YPET (52) have been previously described. *LHY-OX* and *cca1-1 lhy-20* were gifts from Jose Pruneda-Paz. The full-length coding sequence of *ABF3* was driven by the cauliflower mosaic virus 35S promoter to overexpress *ABF3*. ABF3-OX transgenic lines were generated in the *pLHY*::LUC background using Agrobacterium-mediated transformation (floral dip). Arabidopsis seeds were sterilized with 70% ethanol and grown on plates containing ½ MS medium + 0.7% agar (wt/vol) + 1% sugar. After stratification in the dark at 4 °C for 3 d, plates were transferred to 12-h:12-h long-day or 6-h:18-h short-day cycles at 22 °C as indicated. All light conditions were around 80 μmol m^−2^ s^−1^.

### Transcriptome Analysis.

The CCA1-bound genes were obtained from our lab’s published data (45) and the group of Norihito Nakamichi (46), LHY-bound genes were obtained by re-analyzing the data set from the group of Isabelle A. Carre (40). Details were described in *SI Appendix*, *SI Materials and Methods*. The DEG were obtained from the group of Norihito Nakamichi (46). The transcription factors binding the *CCA1* promoter were legacy data of our lab by conducting Y1H screening against the Arabidopsis TF pool (47). Genes overlapping in *cca1 lhy* DEG and three ChIP-seq datasets combined were subjected to Gene Ontology analysis and functional annotation clustering. Each dataset was listed in Dataset S1.

### Bioluminescence Detection.

Transgenic plants expressing *pLHY*::LUC or ABF3-OX/ *pLHY*::LUC were grown on plates containing ½ MS medium + 0.7% agar (wt/vol) + 1% sugar in 12-h:12-h long day for 8 d at 22 °C. Plates were sprayed with 1 mM luciferin and transferred to a plate reader (Tecan Infinite® 200 PRO) under constant white light LL (22 °C, approximately 80 μmol m^−2^ s^−1^) and imaged every hour for at least 5 d. The luminescence data were uploaded and processed with BioDare2 (https://biodare2.ed.ac.uk/) by fast Fourier transform-nonlinear least squares (FFT-NLLS) (65). Statistical analysis and *P* value determination were performed with *t* tests using Excel.

### ChIP Assay.

ChIP assays against ABF3 were performed using *pABF3*::ABF3-YPET (52) with GFP antibody (Thermo Fisher Scientific, cat. #A11122) and ChIP against LHY using Col-0 with LHY antibody (gift from Isabelle A. Carre). Details of the ChIP assay procedure were described in *SI Appendix*, *SI Materials and Methods*.

### Seed Germination.

Ripe seeds were germinated on plates containing ½ MS medium + 0.7% agar (wt/vol) + 1% Sugar with or without NaCl treatment. Plates were placed at 4 °C in the dark for 3 d before transferring to a growth chamber of a 16-h:8-h long-day (22 °C, approximately 80 μmol m^−2^ s^−1^). Radicle protrusion was regarded as seed germination completion. Details of the seed germination assay procedure were described in *SI Appendix*, *SI Materials and Methods*.

### Arabidopsis Gene Identifiers.

Sequence data for genes described in this article can be found in the Arabidopsis Information Resource under the following accession numbers: CCA1 (At2g46830), LHY (AT1G01060), TOC1 (AT5G61380), PRR9 (AT5G02380), ACT7 (AT5G09810), PP2A (AT1G69960), ABF3 (AT3G19290), ABF1 (AT1G59560), ABF2 (AT5G06720), and ABF4 (AT3G19280).

## Supplementary Material

Appendix 01 (PDF)

Dataset S01 (XLSX)

## Data Availability

All study data are included in the article and/or supporting information.
