# Supplementary material for: The interplay between the circadian clock and abiotic stress responses mediated by ABF3 and CCA1/LHY
Source: Proc Natl Acad Sci U S A. 2024 Feb 6;121(7):e2316825121. doi: 10.1073/pnas.2316825121 (PMC10873597; doi:10.1073/pnas.2316825121)
Supplement: Supplementary file 1 — Appendix 01 (PDF) [file pnas.2316825121.sapp.pdf]

**Supporting Information for**

The Interplay between the Circadian Clock and Abiotic Stress Responses Mediated by ABF3 and CCA1/LHY

Tong Liang[a], Shi Yu[a], Yuanzhong Pan[a], Jiarui Wang[a], Steve A. Kay[a,1]

[a]Department of Neurology, Keck School of Medicine, University of Southern California, Los Angeles, CA 90089.

[1]To whom correspondence may be addressed. **Email:** stevekay@usc.edu

**This PDF file includes:**

- Supporting text
- Figures S1 to S3
- Primer List

**Other supporting materials for this manuscript include the following:**

- Transcriptomic Datasets

## Supporting Information

## SI Materials and Methods

**ChIP assay.** For ChIP against ABF3, Arabidopsis seedlings for *pABF3*::ABF3-YPET or Col-0 wild type control were grown on ½ MS plates for 12 d under 8-h:16-h short-day cycles at 22 °C and treated with or without 1 µM ABA for 2 hours before harvesting samples. For ChIP against LHY, Col-0 or *cca1-1 lhy-20* mutant control were grown on ½ MS plates for 12 d under 12-h:12-h long-day cycles at 22 °C before harvesting samples. Two grams of seedlings were harvested and crosslinked with 1% (v/v) formaldehyde (Sigma-Aldrich) for 15 min under a vacuum, and then Glycin was added to quench the crosslink. The seedlings were ground with Mixer mills (Retsch MM400) and the powder was homogenized in nuclear extraction buffer 1 and precipitated by centrifugation at 2000g for 20 min, washed with nuclear extraction buffer 2, and lysed in nuclei lysis buffer. The chromatin was sheared by sonication (Diagenode Bioruptor 300). The chromatin solution was diluted 10-fold with ChIP dilution buffer and incubated with anti-GFP antibody (Thermo Fisher Scientific, cat. #A11122) or anti-LHY antibody (gift from Isabelle A. Carre,) prebound to Dynabeads Protein G (Thermo Fisher Scientific, cat. #10003D) at 4°C. After washing the immunocomplexes, the bound chromatin fragments were eluted with elution buffer and were reversed the crosslink with 200 mM NaCl at 65°C overnight. The genomic DNA was purified with phenol after digesting the proteins with Proteinase-K. Glycogen and NaAc were added to help recover DNA. RT-qPCR was conducted to analyze the immunoprecipitated DNA and input DNA.

**RT-qPCR.** For RT-qPCR, total RNA of seedling plants was isolated using GeneJET Plant RNA Purification Kit (Thermo Scientific, cat. #K0801). cDNA was synthesized from 500 ng of total RNA using a PrimeScript RT reagent kit with gDNA Eraser (Takara, cat. #RR047A). SYBR Greeb Master Mix (applieddbiosystems, cat. #A25742) was used for RT-qPCR on the Real-Time PCR System (Bio-Rad CFX Opus 384). The level of *PP2A* mRNA accumulation (AT1G69960) was used as an internal control. RT-qPCR data for each sample were normalized to the respective *PP2A* expression level. The cDNA was amplified following denaturation via 40 cycles of PCR (95°C, 5 s;

60°C, 20 s per cycle). Three biological replicates and two technical replicates were performed per experiment. Primers are listed in Supplemental Primer List.

**Seed Germination Assays.** Seeds collected at the same time and same growing conditions were used for germination assays. Harvested seeds were air-dried in a silica gel environment at room temperature for 3 weeks before the germination assays. Seeds were sterilized with 70% ethanol and sown on plates containing ½ Murashige and Skoog (MS) medium + 0.7 % agar (wt/vol) + 1% Sugar with or without NaCl treatment. Plates were placed at 4°C in the dark for three days before transferring to a growth chamber with 16-h:8-h long-day (22°C, approximately 80 $\mu mol \cdot m^{-2} \cdot s^{-1}$). Radicle protrusion was regarded as seed germination completion.

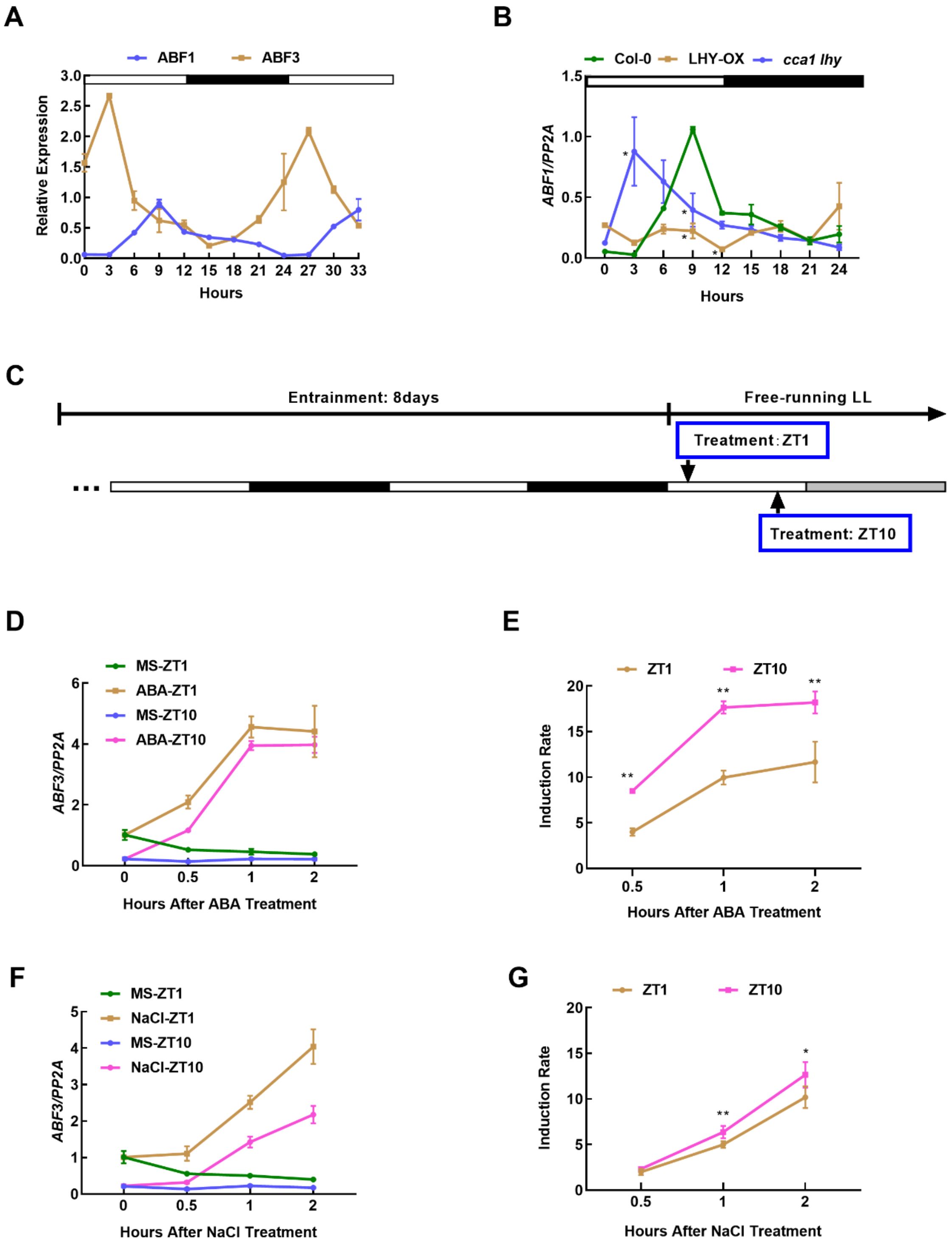

A
ABF1
ABF3
Relative Expression
Hours
B
Col-0
LHY-OX
cca1 lhy
ABF1/PP2A
Hours
C
Entrainment: 8days
Free-running LL
Treatment : ZT1
Treatment: ZT10
D
MS-ZT1
ABA-ZT1
MS-ZT10
ABA-ZT10
ABF3/PP2A
Hours After ABA Treatment
E
ZT1
ZT10
Induction Rate
Hours After ABA Treatment
F
MS-ZT1
NaCl-ZT1
MS-ZT10
NaCl-ZT10
ABF3/PP2A
Hours After NaCl Treatment
G
ZT1
ZT10
Induction Rate
Hours After NaCl Treatment

**Fig.S1.**

(A) *ABF1* and *ABF3* oscillate in the diel condition. RT-qPCR analysis of gene expression of *ABF1* and *ABF3* in wild type (Col-0) in the diel condition. Col-0 was grown in ½ MS under 12-h:12-h long-day cycles for 10 d and collected samples every 3 hours as indicated in the diagram. The *PP2A* gene was analyzed as an internal control. Error bars, SDs of three biological replicates.

(B) The expression of *ABF1* is regulated by LHY and/or CCA1. RT-qPCR analysis of *ABF1* in Col-0, LHY-OX, and *cca1 lhy* in the diel condition. The seeds were grown in ½ MS under 12-h:12-h long-day cycles for 10 d and collected samples every 3 hours as indicated in the diagram. The *PP2A* gene was analyzed as an internal control. Error bars, SDs of three biological replicates. *P < 0.05, compared to corresponding Col-0 of same time point, by t-test using Excel.

(C) The experiment schema for investigating stress-induction at different time points. Col-0 wild type was entrained in ½ MS under 12-h:12-h long-day cycles for 8 d before releasing to long light conditions. The plants were transferred to ½ MS liquid medium containing 50 μM ABA or 120 mM NaCl separately at ZT1 and ZT10, and samples were collected for a time course.

(D) RT-qPCR analysis of gene expression in response to ABA. ABA treatment (50 μM) was applied as indicated in Fig. S1C. The expression of *ABF3* was quantified using *PP2A* gene as an internal control. Error bars, SDs of three biological replicates.

(E) The quantified gene induction rate in response to ABA. The gene induction rate in response to ABA was calculated by dividing ABA with its respective MS medium control at each time point. **P < 0.01 by t-test using Excel.

(F) RT-qPCR analysis of gene expression in response to NaCl. NaCl treatment (120 mM) was applied as indicated in Fig. S1C. The expression of *ABF3* was quantified using *PP2A* gene as an internal control. Error bars, SDs of three biological replicates.

(G) The quantified gene induction rate in response to NaCl. The gene induction rate in response to NaCl was calculated by dividing NaCl with its respective MS medium control at each time point. *P < 0.05, **P < 0.01 by t-test using Excel.

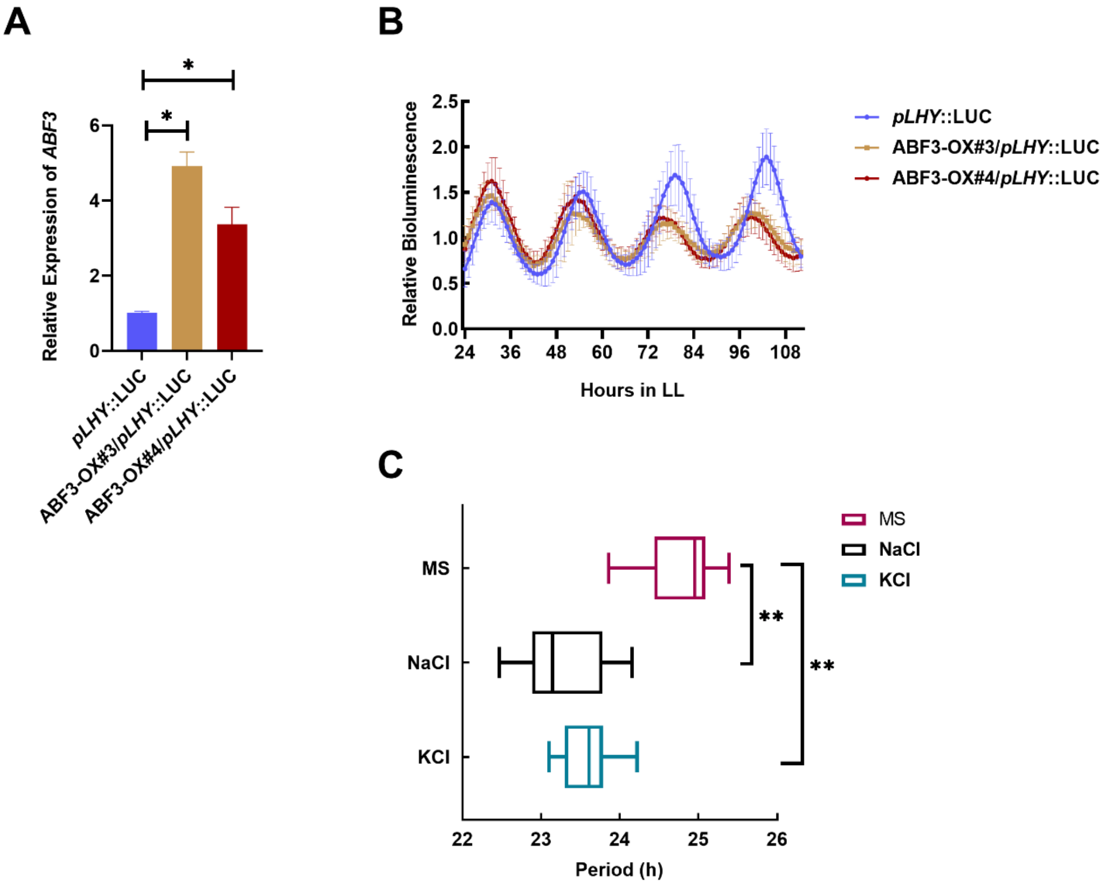


**Fig. S2.**

(A) RT-qPCR verifies the overexpression of *ABF3* in ABF3-OX/ *pLHY*::LUC. The expression of *ABF3* was quantified using *PP2A* gene as an internal control and then normalized to *pLHY*::LUC. Error bars, SDs of three biological replicates. *P < 0.05, by t-test using Excel.

(B) Bioluminescence analysis of *pLHY*::LUC and ABF3-OX/ *pLHY*::LUC. The indicated genotypes were grown in ½ MS under 12-h:12-h long-day cycles for 8d, then released to free-running LL conditions and detected by a bioluminescence reader. Each sample's bioluminescence value of all time points was normalized to its average level. Error bars, mean ± SEM, n = 12.

(C) The circadian period of *pLHY*::LUC, treated with MS medium control, or 120 mM KCl or 120 mM NaCl, as indicated in Fig. 3C. Values are shown as means ± SEM; n = 12. **P < 0.01, by t-test using Excel.

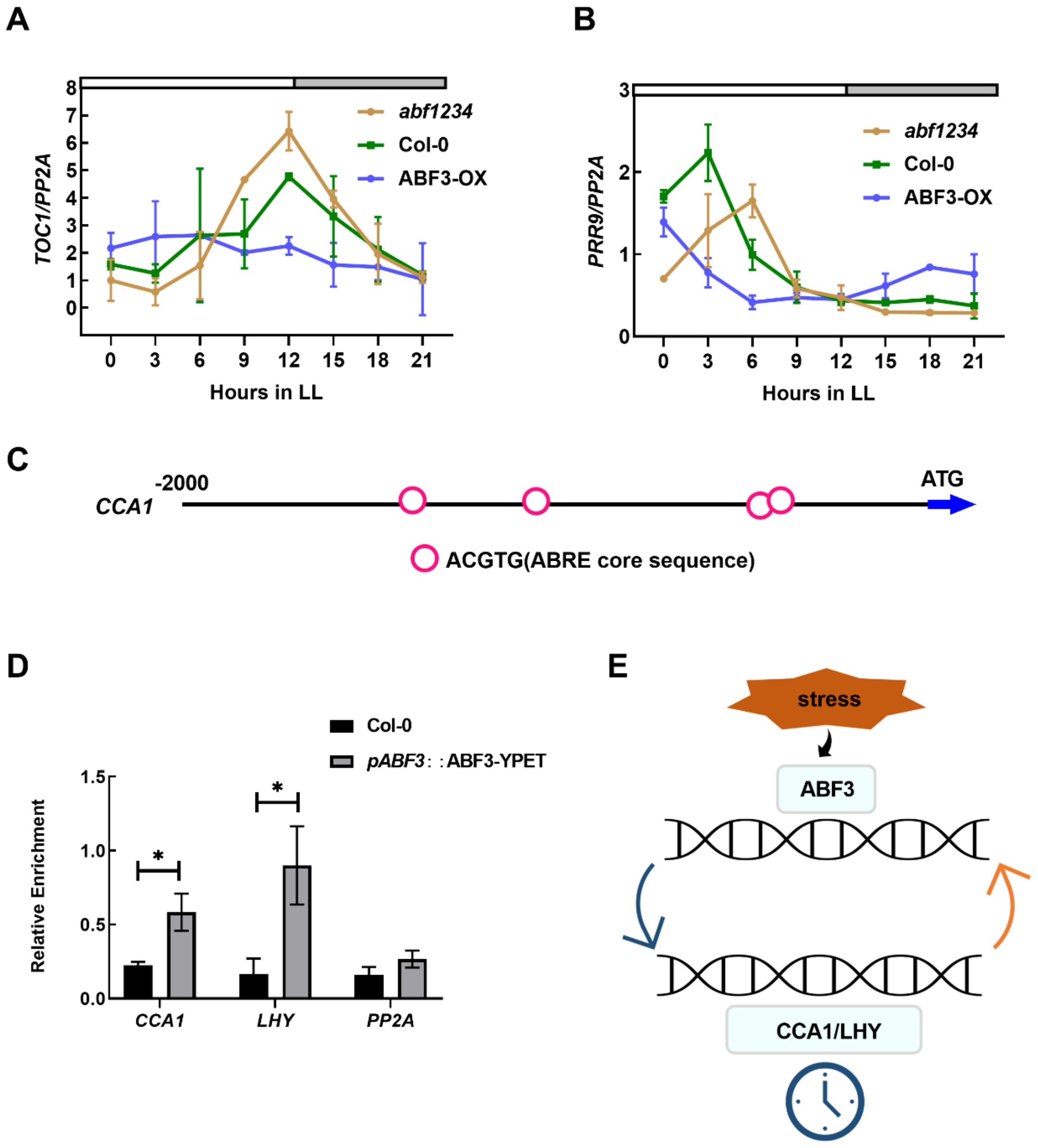


**Fig. S3.**

(A-B) ABF3 regulates the expression of core clock genes in Arabidopsis seedlings. RT-qPCR analysis of gene expression *TOC1* (A), and *PRR9* (B) in Col-0, ABF3-OX and *abf1234*. The indicated genotypes were grown in ½ MS under 12-h:12-h long-day cycles for 10 d, then released to free-running LL conditions. The seedlings were harvested every 3 hours, as indicated in the diagram. The *PP2A* gene was analyzed as an internal control. Error bars, SDs of three biological replicates.

(C) Motif analysis of *CCA1* promoter. The upstream 2000bp of *CCA1* was under analysis. Red circles indicate ACGTG (ABRE core sequence).

(D) ChIP results show that ABF3 binds to the promoters of *LHY* and *CCA1,* instead of *PP2A,* in vivo. 12-day-old Col-0 and *pABF3*::ABF3-YPET transgenic plants were harvested for ChIP assays. Chromatin fragments were immunoprecipitated by anti-GFP beads (IP) or without immunoprecipitation (input). The level of binding was calculated as the ratio between IP and input, normalized to that of *ACT7* as an internal control. Error bars, SDs of three biological replicates. *P < 0.05, by t-test using Excel.

(E) A working model describing the interplay between the circadian clock and abiotic stress mediated by ABF3 and CCA1/LHY.

**Primer List**

| Usage | Name | Primer Pair F/R |
|---|---|---|
| ChIP | ABF3-P1 | GCAATGAAACAGAAATGCGAGA |
| | | AGAAGACCTCACGATGCAAATTA |
| | ABF3-P2 | ACCCTGTTGTTGCAACTTACA |
| | | ACCGGAAGAAACAACACACA |
| | ABF3-P3 | AGGGTCACTTGTATTGTACCGA |
| | | ACTCAATGCCTGCCCTCTTA |
| | ABF3-P4 | TGTTAAGAGGGCAGGCATTG |
| | | CCATTCAAGGGAGCGCATTA |
| | ABF3-P5 | TTGGTGGGAAACGCAAATGT |
| | | GAAAGGGGTCAGAGCGAGAG |
| | ABF3-P6 | CTCACGGCTTTGGATTTGGT |
| | | GCTCACTCACACCATCAACA |
| ChIP | LHY-P1 | CGGTTTGTTTGGTTATTTTGG |
| | | ATCGGTCAGGAAGCCACTAC |
| | LHY-P2 | CCGAACCGAACTAACCAAAG |
| | | CCAGCTTACAACTGTGTTTTGC |
| | LHY-P3 | GTCCAAGCTTGTCGGGTTTA |
| | | TGGTGGTCCACAATTGCTTA |
| | LHY-P4 | TGTGGACCACCACACTCACT |
| | | ATTTGAGGCTGGAACAGCAC |
| | LHY-P5 | TGTGGCTGAGATTGCTTCTG |
| | | TCGTTCCCGGAAATAATCAA |
| | LHY-P6 | TTATTTCCGGGAACGATGAC |
| | | TCTCAGCAGCCAAACAGAGA |
| | LHY-P7 | GTTTGGGTGAGGCTTTTGTG |

| | | |
|---|---|---|
| | | TTGCAAAGCCGTTGTGATAA |
| | LHY-P8 | CGACTGTTTCACGGTGGACT |
| | | TTCGCTGCTTCAAATCCTCT |
| | LHY-P9 | AGAGCGATGGACTGAGGATG |
| | | GGAATCTCCCAATCAAGAGGA |
| ChIP | PP2A | CGGCTTTCATGATTCCCTCT |
| | | GCCTTAAGCTCCGTTTCCTACTT |
| ChIP | ACT7 | CACAATGTTTGGCGGGATTGGTGA |
| | | TGTACTTCCTTTCCGGTGGAGCAA |
| RT-qPCR | LHY | GAC TCA AAC ACT GCC CAG AAG A |
| | | CGT CAC TCC CTG AAG GTG TAT TT |
| RT-qPCR | PRR9 | GCC AGA GAG AAG CTG CAT TGA |
| | | GCT CTG GTA CCG AAC CTT TTT G |
| RT-qPCR | TOC1 | AAT AGT AAT CCA GCG CAA TTT TCT TC |
| | | CTT CAA TCT ACT TTT CTT CGG TGC T |
| RT-qPCR | CCA1 | CCGCAACTTTCGCCTCAT |
| | | GCCAGATTCGGAGGTGAGTTC |
| RT-qPCR | ABF1 | GCTAGATCAAGGGCTCGAAAA |
| | | GGTTTTCATTATTTCAGCCTGTTT |
| RT-qPCR | ABF3 | AATAGGGAATCAGCTGCAAGAT |
| | | GCTTTTCCATGATTTCAACTTGT |
| RT-qPCR | PP2A | TAACGTGGCCAAAATGATGC |
| | | GTTCTCCACAACCGATTGGT |
